# Dipyrone in association with atropine inhibits the effect on gastric emptying induced by hypoglycemia in rats

**DOI:** 10.1590/1414-431X20175948

**Published:** 2017-08-31

**Authors:** E.F. Collares, A.M. Vinagre, C.B. Collares-Buzato

**Affiliations:** 1 Departamento de Pediatria, Faculdade de Ciências Médicas, Universidade Estadual de Campinas, Campinas, SP, Brasil; 2 Núcleo de Medicina e Cirurgia Experimental, Universidade Estadual de Campinas, Campinas, SP, Brasil; 3 Departamento de Bioquímica e Biologia Tecidual, Instituto de Biologia, Universidade Estadual de Campinas, Campinas, SP, Brasil

**Keywords:** Hypoglycemia, Gastric emptying, Dipyrone, Atropine, Muscarinic receptors, Adrenergic receptors

## Abstract

Atropine (AT) and dipyrone (Dp) induce a delay of gastric emptying (GE) of liquids in rats by inhibiting muscarinic receptors and activating β_2_-adrenergic receptors, respectively. The objective of the present study was to determine the effects of pretreatment with AT and Dp, given alone or in combination, on the effect of hypoglycemia in the liquid GE in rats. Male Wistar adult rats (280-310 g) were pretreated intravenously with AT, Dp, AT plus Dp or their vehicle and then treated 30 min later with *iv* insulin or its vehicle (n=8-10 animals/group). Thirty min after treatment, GE was evaluated by determining, in awake rats, the percent gastric retention (%GR) of a saline meal labeled with phenol red administered by gavage. The results indicated that insulin induced hypoglycemia in a dose-dependent manner resulting in a significant reduction in %GR of liquid only at the highest dose tested (1 U/kg). Pretreatment with AT significantly increased %GR in the rats treated with 1 U/kg insulin. Surprisingly, after pretreatment with AT, the group treated with the lowest dose of insulin (0.25 U/kg) displayed significantly lower %GR compared to its control (vehicle-treated group), which was not seen in the non-pretreated animals. Pretreatment with Dp alone at the dose of 40 mg/kg induced an increase in %GR in both vehicle and 0.25 U/kg-treated rats. A higher dose of Dp alone (80 mg/kg) significantly reduced the effect of a marked hypoglycemia induced by 1 U/kg of insulin on GE while in combination with AT the effect was completely abolished. The results with AT suggest that moderate hypoglycemia may render the inhibitory mechanisms of GE ineffective while Dp alone and in combination with AT significantly overcame the effect of hypoglycemia on GE.

## Introduction

The gastrointestinal tract plays an important role in the regulation of post-prandial glycemia and a functionally adequate gastric emptying is fundamental for this to occur (1). Gastric emptying (GE) is a complex process of gastric content transfer to the small intestine, which depends on the integrated action of proximal/distal stomach, pylorus and duodenum, the modulation by central nervous system (CNS), and extrinsic and intrinsic signals of neural, paracrine and endocrine origin (1–7).

A bidirectional relationship between glycemic disturbances and control of GE has been documented in humans (8). Rapid GE, leading to accelerated carbohydrate absorption and increased post-prandial glycemia, may be a contributing risk factor for diabetes and metabolic syndrome in certain ethnic groups (9). Conversely, severe hyperglycemia induces delayed GE in healthy volunteers and diabetic patients (10). Surprisingly, adequate control of glycemia during diabetes is not frequently associated with improvement of GE, suggesting a complex interplay between these two conditions (10). As an example, GLP-1 (glucagon-like peptide 1) agonists, successfully used to control glycemia during diabetes, reduces GE in humans at normal and diabetic states (10,11). Nevertheless, euglycemic hyperinsulinemia has little effect on GE of solids and liquids in diabetes (12).

Insulin- and sustained running-induced hypoglycemia increases GE of liquid in rats (13,14). The mechanisms underlying the effect of reduced blood glucose level on GE are still unclear. Nevertheless, atropine (a nonselective muscarinic antagonist) blocks the increase in liquid GE in the presence of hypoglycemia in humans (15) and rats (16). In rats, the activation of the subtypes M1 and/or M3 muscarinic receptors of smooth muscle fibers of the stomach stimulates GE (16,17) and probably are the subtypes involved in the effect of hypoglycemia on gastric motility.

In clinical practice, dipyrone is a pharmaceutical agent with irrelevant anti-inflammatory activity, but with efficient analgesic, antipyretic and antispasmodic capacities (18). It is considered to be a pro-drug that probably acts through its major metabolites, i.e., 4-methyl-amino-antipyrine (MAA) and 4-amino-antipyrine (AA) (18–20). When administered intravenously (*iv*) to rats, dipyrone delayed GE of a liquid meal (saline) (21,22), an effect that was abolished by electrolytic lesion of the paraventricular nucleus of the hypothalamus and subdiaphragmatic vagotomy (21) and that depended on capsaicin-sensitive afferent fibers (23). Acute *iv* blockade of the release of norepinephrine from peripheral sympathetic nerves with guanethidine or *iv* pretreatment with propranolol (a non-selective β-adrenergic antagonist) abolished the effect of dipyrone on GE, suggesting the involvement of the sympathetic nervous system (SNS), and activation of β_1_- and/or β_2_-adrenergic receptors during the phenomenon (24).

The sympathetic-adrenal system is rapidly activated in order to increase blood glucose levels in hypoglycemia and the α_2A-_receptor is an important regulator of glycemia which, when activated, inhibits the release of insulin by pancreatic β cells (25). It has been recently suggested that activation of peripheral β_2_-adrenoceptors (possibly in the liver) and of the ventromedial region of the hypothalamus also occurs in rats with insulin-induced hypoglycemia as a counter-regulatory response for the recovery of glycemic levels (26,27).

Although there is no evidence that the activation of the sympathetic-adrenal system during hypoglycemia interferes in gastric motor function, it has been shown that systemically administered dipyrone induces delayed GE by activating β_2_-adrenoceptors (28). Therefore, it is tempting to hypothesize that this phenylpyrazolone derivative may block or reduce the effect of hypoglycemia on GE. On this basis, the objective of the present study was to investigate the effect of pretreatment with atropine and dipyrone, separately or combined, on the effect of hypoglycemia on liquid GE in rats.

## Material and Methods

Male Wistar rats weighing 280-310 g were obtained from breeding colonies of the Centro Multidisciplinar para Investigação Biológica na Área da Ciência em Animais de Laboratório (CEMIB) of the Universidade Estadual de Campinas (UNICAMP, Brazil). After adaptation to laboratory conditions for 2 weeks, the animals were housed in individual cages with free access to food and water, which were removed 24 h and 30 min, respectively, before the animal studies that started between 1:00 and 4:00 pm.

Solutions of dipyrone (that were protected from light) and atropine sulfate (both from Sigma, USA) and regular swine insulin (Neosulin-Biobras, Brazil) were prepared at the time of use in sterile saline.

At the beginning of the study, the animals received the following pretreatments *iv*: 1) atropine sulphate (at two doses, 1 or 5 mg/kg, AT1 or AT5 groups, respectively); 2) dipyrone (at two doses, 40 or 80 mg/kg, Dp40 or Dp80 groups, respectively), 3) a mixture of atropine (1 mg/kg) and dipyrone (40 or 80 mg/kg, M40 or M80 groups) or vehicle (saline 1 mL/kg, C group). After 30 min, each pretreated group was treated *iv* with vehicle (V; saline, 1 mL/kg weight), 0.25 U/kg insulin (0.25 U) or 1 U/kg insulin (1 U) Firstly, we investigated the effect of atropine alone in order to determine the most effective dose of this drug to be used in the subsequent steps of the study (n=8 animals/treated group). In the second part of this work, we concomitantly evaluated the effect of pretreatment with dipyrone alone or in combination with atropine, followed by respective treatment with vehicle or insulin at the two different doses tested (n=10 animals/treated group).

Thirty min after treatment, with the animal awake, we determined the GE of a saline test liquid meal labeled with phenol red (60 µg/mL). The test meal was given by gavage in a volume of 2 mL/100 g body weight and 10 min after its administration the percentage of gastric retention (%GR) was determined using a standard technique (29) with two modifications: 1) the reading for determination of concentration of phenol red dye was done by spectrophotometry at 560 nm, and 2) before euthanasia, the animals were sedated with halothane (23).

At the end of the procedure for %GR determination, before the animals were euthanized, blood was collected from the abdominal aorta into an appropriate flask (in fluoride/EDTA, 2 mL) for the measurement of plasma glucose (reported as mg/dL) using the o-toluidine method (30).

All experimental procedures were approved by the Ethics Committee for Animal Experimentation (CEEA/UNICAMP, protocol No. 1372-1).

Data were analyzed statistically by ANOVA followed by the Tukey post-test, with the significance limit set at α = 0.05 for both tests.

## Results

**Figure 1.**
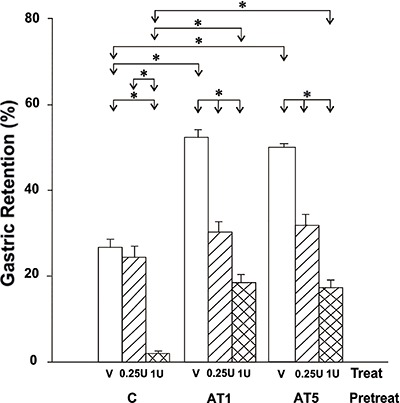
Gastric retention of a saline test meal (2 mL/100 g animal weight), 10 min after administration by gavage to rats. The animals were pretreated (Pretreat) *iv* with saline (C), 1 or 5 mg/kg atropine sulfate (AT1 and AT5, respectively) and, after 30 min, treated (Treat) *iv* with vehicle (V), 0.25 U or 1 U/kg insulin (0.25U and 1U, respectively). The test meal was administered 30 min after treatment. Data are reported as means±SE for 8 animals per group. *P<0.05 (ANOVA followed by Tukey post-test).

As shown in Table 1, insulin significantly reduced plasma glucose concentration in a dose-dependent manner (0.25 U/kg; 1 U/kg) while the pretreatment with 1 or 5 mg/kg atropine did not significantly modify the glucose levels of the treated groups compared to their controls (C+V, C+0.25 U, and C+1 U, respectively). The insulin-induced hypoglycemia resulted in significant reduction in %GR of liquid only at the highest dose tested (1 U/kg) compared to the control group (C+V). Pretreatment with atropine at concentrations of 1 or 5 mg/dL significantly increased %GR in treated groups with saline (AT1+V and AT5+V) or with insulin 1 U/kg (AT1+1 U and AT5+1 U) compared to non-pretreated ones (C+V and C+1 U, respectively). Regardless of the atropine concentration used, pretreatment with this muscarinic antagonist reduced the effect of 1 U/kg insulin on %GR since the pretreated rats displayed a decrease of about 64% in this parameter (AT1+1 U *vs* AT1+V and AT5+1 U *vs* AT5+V) in contrast with the non-pretreated (C+1 U) ones that showed a 91% reduction in %GR compared to their respective control (C+V). After pretreatment with atropine at both concentrations, the groups treated with the lowest insulin dose displayed significantly lower %GR compared to its control groups (AT1+V and AT5+V; Figure 1).

**Table 1.** Plasma glucose (mg/dL) levels of rats 30 min pretreated *iv* with saline (C), 1 or 5 mg/kg atropine sulfate (AT1 and AT5, respectively) and then *iv* treated with vehicle (V), 0.25 U or 1 U/kg insulin (0.25 U and 1 U, respectively).

Treatment/Pretreatment	V	0.25 U	1 U
C	98.1±6.1	65.7±0.7^a^	33.3±1.4^a^,^d^
AT1	113.7±3.5	77.6±4.6^b^	37.1±2.4^b^,^e^
AT5	115.1±4.6	65.7±2.7^c^	31.9±1.8^c^,^f^

Data are reported as means±SE of 8 rats.

a,b,c P<0.05 *vs* C+V, AT1+V, and AT5+V, respectively;

d,e,f P<0.05 *vs* C+0.25, AT1+0.25, and AT5+0.25 U, respectively, (ANOVA followed by Tukey post-test).

**Figure 2.**
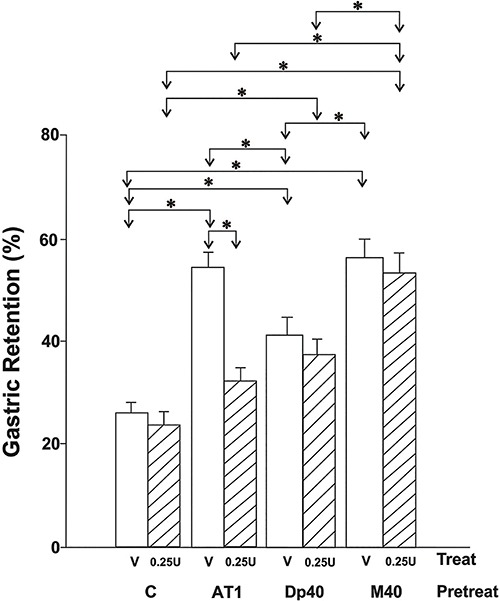
Gastric retention of a saline test meal (2 mL/100 g animal weight), 10 min after administration by gavage to rats. The animals were pretreated (Pretreat) *iv* with saline (C), 1 mg/kg atropine sulfate (AT1), 40 mg/kg dipyrone (Dp40), or a mixture of 1 mg atropine+40 mg/kg dipyrone (M40), and after 30 min, treated (Treat) *iv* with vehicle (V) or 0.25 U/kg insulin (0.25U). The test meal was administered 30 min after treatment. Data are reported as means±SE for 10 animals per group. *P<0.05 (ANOVA followed by Tukey post-test).

**Figure 3.**
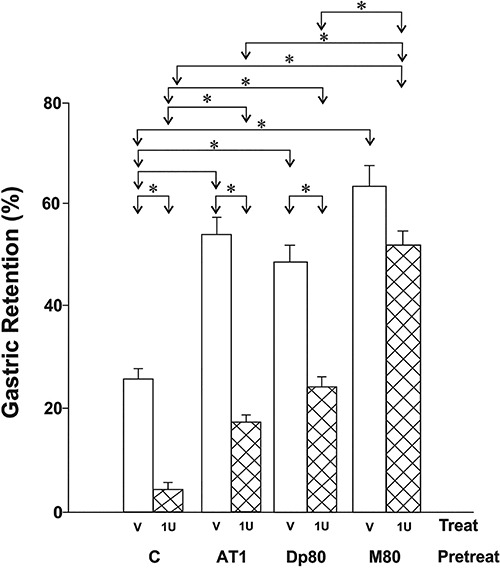
Gastric retention of a saline test meal (2 mL/100 g animal weight), 10 min after administration by gavage to rats. The animals were pretreated (Pretreat) *iv* with saline (C), 1 mg/kg atropine sulfate (AT1), 80 mg/kg dipyrone (Dp80), or a mixture of 1 mg atropine+80 mg/kg dipyrone (M80) and, after 30 min, treated (Treat) *iv* with vehicle (V) or 1 U/kg insulin (1U). The test meal, was administered 30 min after treatment. Data are reported as means±SE for 10 animals per group. *P<0.05 (ANOVA followed by Tukey post-test).

In the second part of the study, since the effects of atropine on %GR were independent of concentrations, we employed only the 1 mg/kg dose of atropine in the pretreatment experiments testing the combination with dipyrone (Figures 2 and 3).

The plasma glucose concentrations of the animals pretreated with saline (C), 1 mg/kg atropine (AT1), 40 mg/kg dipyrone (Dp40) or with a mixture of both components (M40) and followed by the treatment with saline (V) or with 0.25 U/kg insulin (0.25 U) are reported in Table 2 and the %GR data in Figure 2. Insulin significantly reduced glucose levels in treated animals regardless of the pretreatment applied, while the pretreatment with dipyrone alone or in combination with atropine did not affect glycemia of the animals.

**Table 2.** Plasma glucose levels (mg/dL) of rats pretreated *iv* with saline (C), atropine sulfate (1 mg/kg AT1), dipyrone (40 mg/kg, Dp40; or 80 mg/kg, Dp80), or with a mixture of atropine (1 mg/kg) and dipyrone (M40=AT1+Dp40 mixture, or M80=AT1+Dp80 mixture) followed by the *iv* treatment with vehicle (V), 0.25 U or 1 U/kg insulin (0.25 U and 1 U, respectively).

Treatment/Pretreatment	V	0.25 U	1 U
C	99.0±5.0	63.9±1.4^a^	33.2±1.9^a^,^g^
AT1	108.2±4.7	74.6±4.2^b^	34.6±2.5^b^,^h^
Dp40	108.3±5.5	67.7±2.8^c^	nd
M40	100.8±2.8	76.5±3.4^d^	nd
Dp80	109.2±3.1	nd	42.2±2.9^e^,^g^
M80	116.0±5.6^i^	nd	44.0±2.2^f^,^h^

Data are reported as means±SE of 10 rats.

a,b,c,d,e,f P<0.05 *vs* C+V, AT1+V, Dp40+V, M40+V, Dp80+V, and M80+V, respectively;

g,h P<0.05 *vs* C+0.25 and AT1+0.25 U, respectively;

i P<0.05 *vs* C+V (ANOVA followed by Tukey post-test). nd: not determined.

As shown previously in Figure 1, the moderate hypoglycemia induced by insulin at a relatively low dose (i.e., 0.25 U/kg) did not result in significant changes in %GR compared to vehicle-treated controls (C+V group). In contrast, atropine-pretreated rats displayed significant reduction in %GR after insulin injection (AT1+0.25 U group) compared to their vehicle-treated controls (AT1+V group) (Figure 2). Pretreatment with dipyrone (Dp40) increased %GR in the hypoglycemic animals (Dp40+0.25 U group) compared to C+0.25 U group but did not differ significantly from that observed in the normoglycemic animals with the same pretreatment (Dp40+V group).

The same phenomenon was observed in the hypoglycemic animals pretreated with the mixture (M40+0.25 U group), with higher %GR values compared to C+0.25 U group and that did not differ significantly from those observed in the animals pretreated with the mixture and treated with V (M40+V group; Figure 2).

Table 2 also presents the plasma glucose values of the animals pretreated with 1 mg/kg atropine (AT1), 80 mg/kg dipyrone (Dp80) or with the mixture of both drugs (M80) and treated with vehicle (V) or 1 U/kg insulin (1 U). Figure 3 presents the %GR values under the same pretreatment and treatment conditions.

Insulin at the dose of 1 U/kg markedly reduced the plasma glucose levels of treated animals regardless of the pretreatment applied. Meanwhile, pretreatment with either atropine or dipyrone did not affect significantly the glycemia in rats. Nevertheless, in the group pretreated with the mixture of atropine and dipyrone followed by the vehicle administration (M80+V group), plasma glucose showed a subtle but significant increase compared to the group that only received saline as a pretreatment (C+V group).

Concerning the GE data, pretreatment with dipyrone alone significantly increased %GR in the animals that received only vehicle (Dp80+V group) compared to those non-pretreated (C+V group). Meanwhile, pretreatment with dipyrone partially inhibited the effect of treatment with insulin (Dp80+1 U group) on %GR: while the C+1 U group showed a decrease of 84% in %GR compared to the C+V group, this reduction was only 49% in Dp80+1 U group compared to its respective Dp80+V group. This effect of dipyrone was potentiated by the presence of atropine (M80), which significantly abolished the effect of hypoglycemia on %GR in rats (Figure 3).

## Discussion

As mentioned previously, GE is a very complex process. From the functional point of view, the proximal stomach (the fundus and most of the gastric body) presents tonic activity that accommodates the food bolus and propels the contents to the distal stomach through subtle contractions that overlap the tonus and increase the intraluminal pressure (3,4). The processes that accommodate the ingested material without substantial increase of the intragastric pressure involves inhibitory pathways mediated by the dorsal vagus complex, the activation of which leads to: 1) the receptive relaxation mediated by vago-vagal reflex and triggered by swallowing, and 2) the accommodation mediated by the activation of gastric mechanoreceptors, which are stimulated when the stomach is distended, triggering intrinsic and vago-vagal reflexes (3,7,31). The neurotransmitters of the gastric accommodation are nitric oxide and vasoactive intestinal peptide (3,7,31).

The distal stomach, constituted by a small part of the body and antrum, presents peristaltic activity responsible for grinding the solids, mixing and propulsion of the contents through the pylorus that only allows the passage of very small solid fragments to the duodenum during GE (3,4). Acetylcholine and tachykinins are involved in the excitatory transmission (3,5,32,33). Yet, the regulation of GE involves receptors within the intestinal wall that seem to respond to osmolarity and chemical composition (H+, lipids and proteins) of the intestinal luminal content; when these receptors are stimulated, this results in the release of gastrointestinal hormones and factors (3,4,34). The participation of the SNS in the physiological mechanisms that regulate the motor activity of the stomach is presumably negligible compared to vagal influences, although in situations of stress it may contribute to the reduction of gastric motility (3).

The site(s) and mechanism(s) underlying the effect of circulating glucose concentration on GE are still unclear. Most studies investigate GE at a hyperglycemic state, yielding some contradictory results (34–37). It has been proposed that glucose acts in the hepatic portal area to inhibit hepatic afferent nerve traffic to the CNS, which results in a depression reflex in intragastric pressure (35) and, as consequence, a decrease in GE. In contrast, another study suggested that the primary site of the inhibitory action of glucose on gastric motility is the CNS, probably the dorsal motor nucleus of the vagus (DMV), rather than the periphery (36). According to these authors, glucose would predominantly inhibit preganglionic excitatory neurons that make synapses with myenteric cholinergic neurons (37), slowing GE.

The mechanism underlying the effect of glucose deprivation on GE is even less clear. It has recently been suggested that astrocytes attached to the nucleus tractus solitarius (NTS), in the dorsal hindbrain, are involved in triggering processes that affect gastrointestinal motility during glucose deprivation (38). According to this hypothesis, a cytoglucopenia in astrocytes would release an inhibitory gliotransmitter (probably adenosine) to NTS neurons resulting in inhibition of the gastric-NTS neurons. Consequently, there is a decrease in the activity of the inhibitory gastric neurons and disinhibition of the excitatory gastric neurons of the DMV resulting in increased gastric motility during hypoglycemia (38).

In the present study, our data showing that atropine inhibited the acceleration of GE induced by hypoglycemia after a high dose of insulin (1 U/kg) suggested that muscarinic receptors were activated by acetylcholine released in the hypoglycemic condition, corroborating the above hypothesis and confirming previous observations in humans and rats (15,16).

It is known that atropine reduces gastrointestinal motility (39), probably inducing a delay of GE through a predominance of inhibitory mechanism of the motor activity of the stomach as a consequence of the blockade of muscarinic receptors. This phenomenon was identified in the present study since %GR of a non-caloric isotonic liquid meal was significantly greater in normoglycemic animals pretreated with atropine compared to their vehicle-treated controls. Interestingly, we observed that in animals treated with a lower insulin dose (0.25 U/kg), in which hypoglycemia was not so marked, atropine pretreatment resulted in a significantly lower %GR compared to normoglycemic animals pretreated with this cholinergic antagonist. In contrast, in animals with a more conspicuous reduction of plasma glucose, atropine “normalized” the GE of the test meal, although the %GR values of the hypoglycemic group did not reach the level of the normoglycemic group that received the same drug.

These observations suggested a differential effect of hypoglycemia on the GE of liquid depending on the degree of the reduction in plasma glucose level. The relatively mild hypoglycemia, induced by low dose of insulin, may have rendered the inhibitory pathways of GE inoperative, apparently without activating stimulatory pathway. This attenuation of the GE inhibitory inputs, induced by mild hypoglycemia, was only evident when the stimulatory (cholinergic) pathway was blocked by atropine pretreatment (which caused an imbalance in GE regulatory process). Meanwhile, the relatively rapid GE induced by a marked hypoglycemia after high insulin dose is probably a result of the combination of two events: attenuation of the inhibitory pathways and simultaneous activation of muscarinic stimulatory receptors. Overall, our atropine pretreatment data are in line with the idea mentioned above of an inhibition of the gastric vagal reflex circuits within the DMV triggered by the glucopenia of NTS astrocytes to explain the repercussion of the hypoglycemia on GE (38).

Concerning dipyrone, it has been reported that this drug induces delay of GE in rats (21,22), which was confirmed herein. Although the mechanism is still unknown, a recent study using selective β-antagonists and surgical sympathectomy suggested the participation of β2-adrenoceptors in the GE reduction induced by this drug, even though the peripheral origin of the neurotransmitter remained undetermined (28).

In the present study, GE of hypoglycemic rats after pretreatment with dipyrone at the dose of 40 mg/kg, in combination or not with atropine (M40+0.25 and Dp40+0.25 U, respectively) was similar to that seen in their respective normoglycemic control group (M40+V or Dp40+V) but significantly higher compared to the GE of those that were not pretreated with this drug (C+0.25 U). Interestingly, the relatively lower GE, evidenced after pretreatment with atropine in 0.25 U insulin-treated rats, was not seen when this muscarinic inhibitor was co-administered with dipyrone. This result indicated that the pretreatment with dipyrone may overcome the attenuation or inactivation of the inhibitory mechanisms of GE determined by moderate hypoglycemia (insulin at the dose of 0.25 U/kg).

Regarding the animals with more pronounced hypoglycemia, pretreatment with dipyrone (80 mg/kg) alone significantly inhibited, but not completely, the acceleration of GE seen in these rats in relation to their control. The total blockage of the hypoglycemia-induced GE increase was achieved with 80 mg/kg dipyrone in combination with atropine, indicating that, in the latter condition, the inoperativeness of the inhibitory mechanisms and the stimulation of the muscarinic pathway were overcome concomitantly.

Since the effects of pretreatment with dipyrone and atropine on GE at the hypoglycemic condition were additive, it is plausible to suggest that these drugs acted on independent pathways that affect GE. However, since it is well known that there are complex and multilevel interactions between parasympathetic and sympathetic nervous systems (40), it is possible that the effects of dipyrone and hypoglycemia on GE may have some degree of interplay. In addition, pretreatment with the drugs tested here did not cause significant changes in plasma glucose, except for the M80+V group, in which a subtle but significant elevation of this parameter was observed for an unknown reason. Therefore, taken together, these results suggest that the effects of atropine and dipyrone on GE were not a result of their action on plasma glucose level.

Dipyrone, the most popular pyrazolone derivative is used as an antipyretic and analgesic in some countries (18). Our work in rats may suggest that this drug should be used with caution in diabetic patients due to its effect on GE. Nevertheless, additional study is needed since the effect on GE in humans is still unknown.

In conclusion, our study shed light on the intricate mechanism involved in the regulation of GE by glycemia. Our data suggested that dipyrone alone and in combination with atropine, without significantly changing blood glucose levels, overcame the effect of hypoglycemia on the GE of a liquid meal in rats, probably activating the β2-adrenergic inhibitory and counter-interacting muscarinic stimulatory pathways, respectively.
